# Relationship between Air Pollutants and Economic Development of the Provincial Capital Cities in China during the Past Decade

**DOI:** 10.1371/journal.pone.0104013

**Published:** 2014-08-01

**Authors:** Yunpeng Luo, Huai Chen, Qiu'an Zhu, Changhui Peng, Gang Yang, Yanzheng Yang, Yao Zhang

**Affiliations:** 1 State Key Laboratory of Soil Erosion and Dryland Farming on the Loess Plateau, College of Forestry, Northwest A&F University, Yangling, Shaanxi, China; 2 Chengdu Institute of Biology, Chinese Academy of Sciences, Chengdu, China; 3 Center of CEF/ESCER, Department of Biology Science, University of Quebec at Montreal, Montreal, Canada; Chinese Academy of Sciences, China

## Abstract

With the economic development of China, air pollutants are also growing rapidly in recent decades, especially in big cities of the country. To understand the relationship between economic condition and air pollutants in big cities, we analysed the socioeconomic indictorssuch as Gross Regional Product per capita (GRP per capita), the concentration of air pollutants (PM_10_, SO_2_, NO_2)_ and the air pollution index (API) from 2003 to 2012 in 31 provincial capitals of mainland China. The three main industries had a quadratic correlation with NO_2_, but a negative relationship with PM_10_ and SO_2_. The concentration of air pollutants per ten thousand yuan decreased with the multiplying of GRP in the provinical cities. The concentration of air pollutants and API in the provincial capital cities showed a declining trend or inverted-U trend with the rise of GRP per capita, which provided a strong evidence for the Environmental Kuznets Curve (EKC), that the environmental quality first declines, then improves, with the income growth. The results of this research improved our understanding of the alteration of atmospheric quality with the increase of social economy and demonstrated the feasibility of sustainable development for China.

## Introduction

China has seen economic soaring in the past three decades, with its gross domestic product (GDP) expanding 140 times during 1978–2012 (National Bureau of Statistics, 2013). However, such economic soaring is accompanied with deterioration of the atmospheric quality. In the first three months of 2013, just like what happened in London in 1952 [1], long-time haze influenced large area of China (Fig S1), which further stimulated the strong demand for improvement of air quality.

Air pollution has significant influence on both climate and human health [2], [3]. Oxidising air pollutants like ozone stimulate reactions to produce more greenhouse gases which exacerbate global warming [4]. Besides, decreasing precipitation and increasing dimness [5], [6], widening of the tropics [7], weakening of summer moonsoon in South Asian [4], [8], as well as large-scale ocean circulation and some extreme weather like hurricane [9], [10], are all linkd to air pollution. Moreover, anthropogenic air pollutants, especially particulate matter is extremely harmful to human health. According to Silva *et al*. (2013), more than 2 million premature deaths are associated with PM_2.5_-related diseases [11]. Research results from Spain and England reported that long-term exposure to air pollution mainly explained heart disease morbidity and mortality [12], [13]. Similarly, the heating policy in Northern China was found to cause reduction in life expectancies of Northern residents by about 5.52 years [14]. Given the great influece of air pollution on natural environment and human life, researches are attaching ever greater importance to the causes and effects of air pollution [4], [15]–[17].

Environmental problems result from economic expansion which increases extraction of natural resources and accumulation of waste, in the end exceeding the carrying capacity of the biosphere to the pollutants [18]. From the perspective of the history of human society economy development, the environmental quality is not fixed along a country's development path [19]–[21]. In the 1990s, scientists found an inverted U-shaped relationship between environmental quality and social income [22]–[25]. Such relationship was defined as Environmental Kuznets Curve (EKC), showing that the environmental quality would first deteriorate with the increase in revenue, and then it would improve when incomes rise to a certain level [25]. Numerous research results related to developed countries have identified EKC curve between income and air pollutants, especially in the Organization for Economic Co-operation and Development (OECD) countries [18], [23], [26]. However, the relationship between income and air pollutants varies considerably among developing countries. For example, the same air pollutant sulfur dioxide (SO_2_) showed an inverted U-shape relationship with income for Tunisia, but an N-shape relationship for Turkey [27], [28].

China is the biggest developing country in the world, whose high-speed economic development as well as environmental changes and protection may provide experiences and lessons for other developing countries in this respect. There are also some studies regarding EKC in China [29], [30]. However, most of them focused on econometrics, without relating air pollutants to specific levels of economic development. Considering the disparate economy development paces of different provinces in this big country, analysis about how particular air pollution is related to economic development of each region is needed [31]. Moreover, EKC researches in China concentrated on comprehensive indictors like the total amount of atmospheric emission [32]–[35]. Although some researches studied specific pollutants like SO_2_ or PM [36]–[37], the relationship between the most important three categories of air pollutants and socioeconomic indicators is not adequately reported.

In this study, we aimed to establish regression models to fit the relationship between air pollutants and the three major industries (the primary, secondary and tertiary industries), so as to reveal the relationship between industry and air quality deterioration. We also caculated the ratio of air pollutant concentration to Gross Regional Product (GRP) per capita in order to know the contribution of economic development to air pollution over time. Finally, regression analysis was conducted to verify the existence of EKC in Chinese cities, or to define the otherwise relationship between air pollutants and revenue of Chinese citizens.

## Datasets and Methods

### 1. Study area and data source

Data were collected for 31 provincial capitals in mainland China, which are representative of the general condition of each province (Fig. 2). In order to investigate the relationship between social economy and concentration of environmental pollutants in China, we downloaded data about these two aspects in the database of the National Bureau of Statistics of China (Table S1). The economic data included GRP, population, primary industry output, secondary industry output and tertiary industry output. The pollutant data collected included the concentration of PM_10_, SO_2_ and NO_2_ (the three most important air pollutants in China [38]). The air pollution index (API) was calculated with the following formula:
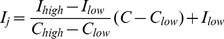
Where:

**Figure 1:**
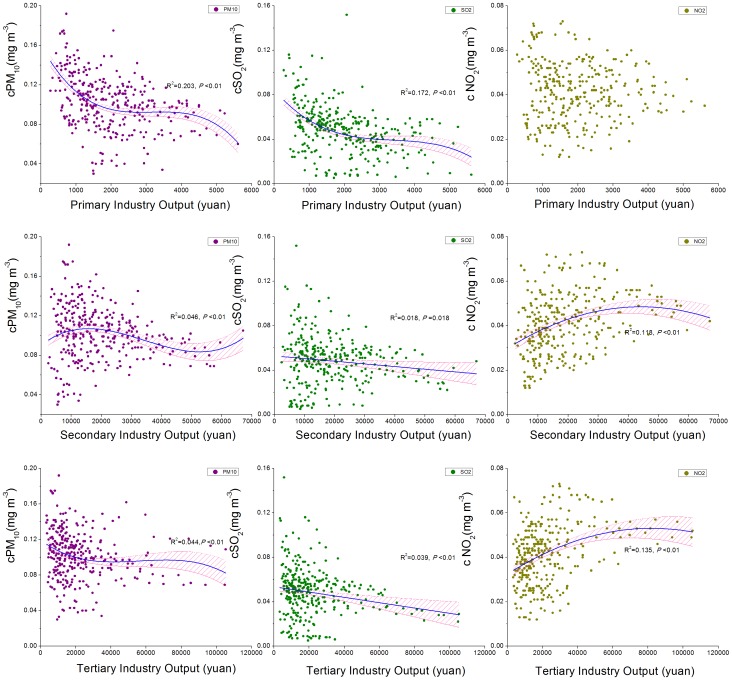
Air pollutant concentrations as related to the output per capita of three industries in the provincial capitals of China. (a) The output per capita of the primary industry; (b) The output per capita of the secondary industry; (c) The output per capita of the tertiary industry.

**Figure 2:**
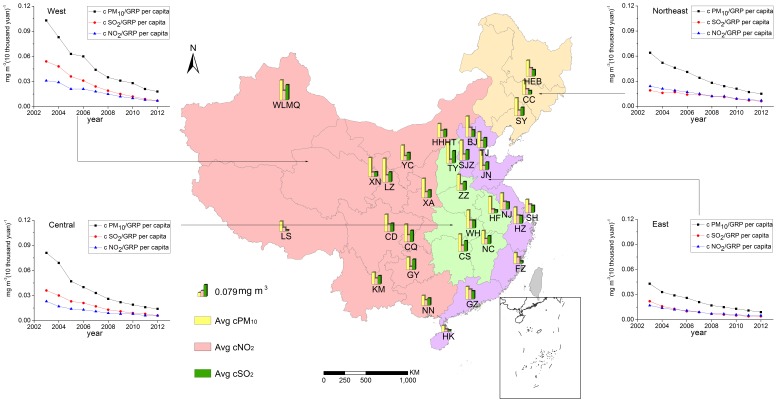
Annual mean concentration of PM_10_, SO_2_ and NO_2_ from 2003 to 2012 in different province capitals of mainland China (bar charts on the Chinese map). Four line charts represent the relationships between annual mean air pollutant and GRP per capita of the East, Central, Northeast and West China respectively from 2003 to 2012.

*I* = (Air pollution) index of one specific pollutant,

*C* = pollutant concentration,

*C*_low_ = the concentration breakpoint ≤C,

*C*_high_ = the concentration breakpoint ≥C,

*I*_low_ = the index breakpoint corresponding to C _low_,

*I*_high_ = the index breakpoint corresponding to C _high_,

j = Air pollutants indicators (PM_10_, SO_2_, NO_2_).
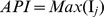



The criteria of breakpoints for air pollutants were taken from the website of Ministry of Environment Protection of the People's Republic of China [39].All data from 2003 to 2012 used for statistical analyses were retrieved from National Bureau of Statistics of China. As the demographic data for Lhasa during 2003–2006 and 2010 was missing, we did not do analyze the city for these years.

### 2. Relationship between air pollutants and the three main industries

Linear, quadratic and cubic regression analysis was conducted to examine whether there existed simple positive or negative relationship between the concentration of air pollutants (dependent variables) and the output per capita of the three industries (independent variables) in the provincial capital cities. We chose the best appropriate regression model for each air pollutant and industry and plotted the regression line for those which were significantly correlated.

### 3. Trend analysis for the ratio of pollutant concentration to industry output

For comparing socioeconomic development level in different regions in China, we classified all the 31 provincial capital cities of mainland China into four economic regions including East Coast (East), Central China (Central), Northeastern China (Northeast), and Western China (West), according to strategies promulgated by the Central People's Government [40]. The ratio of annual air pollutant concentration to GRP per capita (c PM_10_/GRP per capita, c SO_2_/GRP per capita, and c NO_2_/GRP per capita) was calculated year by year for each region. Hereafter, the line trend plots of the ratios were constructed to illustrate the variation of energy efficiency during 2003 to 2012.

### 4. Analysis associated with EKC

In order to investigate whether EKC exists in China, regression methods were applied to the panel data of GRP per capita and pollutants' indicators in all provincial capital cities. We also conducted regression fitting for the four economic regions for further information. The relationships between air pollutants and GRP per capita were estimated by the simplified EKC model provided below, which was also described by Agras et al. (41) and Li et al. (42)[41]–[42]:
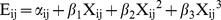
Where E is the concentration of air pollutant; X is GRP per capita; α_ij_ is a fixed effect; ε_ij_ is a stochastic error term; i is a region index (region values are “East, Central, Northeast, West and All provincial capital cities”); j is an air pollutant indicator (PM_10_, SO_2_, NO_2_ or API); β_1_, β_2_, β_3_ are the coefficient for the income variable, for the income squared variable and for the income cubic term, respectively.

### 5. Statistical analysis

All the regression analysis related to air pollutants and socioeconomic indicators was performed with SPSS for Windows (IBM SPSS statistics; Version 20). The effect of a certain variable was considered statistically significant for P<0.05. Annual mean values of data used for trend analysis of energy effiency between 2003 to 2012 were caculated by Excel 2010.

## Results

### 1. Relationship between air pollutants and the three main industries in Chinese cities

Analysis on the provincial capital cities illustrated quadratic relationships between concentration of NO_2_ and the output per capita of secondary and tertiary industries (Table 1, Fig. 1). The NO_2_ concentration rose with the increase of the output per capita of the secondary and tertiary industries at the first stage, then began to decrease when the output reached around 45,000 and 70,000 yuan respectively. However, there was no remarkable relationship between that of the primary industry and the NO_2_ concentration. The results also indicated that all the three industries had significantly negative relationship with the concentration of PM_10_ and SO_2_.

**Table 1:** Regression for concentration of PM_10_, SO_2_, NO_2_ and the three main industries.

	Model summary						
	Regression					Coefficients T test	
Described Relationship	Model	n	R^2^	SE	Sig.	Independent variable	Constant
PM_10_	Linear		0.147	0.026	0.000^**^	0.000^**^	0.000^**^
&	Quadratic		0.178	0.025	0.000^**^	0.000^**^	0.000^**^
Primary industry	Cubic		0.203	0.025	0.000^**^	0.000^**^	0.000^**^
SO_2_	Linear		0.141	0.020	0.000^**^	0.000^**^	0.000^**^
&	Quadratic	305	0.163	0.020	0.000^**^	0.000^**^	0.000^**^
Primary industry	Cubic		0.172	0.020	0.000^**^	0.001^**^	0.000^**^
NO_2_	Linear		0.004	0.013	0.280		
&	Quadratic		0.007	0.013	0.348		
Primary industry	Cubic		0.016	0.013	0.172		
							
PM_10_	Linear		0.025	0.027	0.005^**^	0.005^**^	0.000^**^
&	Quadratic		0.034	0.027	0.006^**^		
Secondary industry	Cubic		0.046	0.027	0.003^**^	0.035*	0.000^**^
SO_2_	Linear		0.018	0.022	0.018*	0.018*	0.000^**^
&	Quadratic	305	0.022	0.022	0.041*	0.873	0.000
Secondary industry	Cubic		0.021	0.022	0.092		
NO_2_	Linear		0.097	0.012	0.000^**^	0.000^**^	0.000^**^
&	Quadratic		0.118	0.012	0.000^**^	0.000^**^	0.000^**^
Secondary industry	Cubic		0.118	0.012	0.000^**^	0.100^**^	0.000^**^
							
PM_10_	Linear		0.029	0.027	0.003^**^	0.003^**^	0.000^**^
&	Quadratic		0.037	0.027	0.003^**^	0.012*	0.000^**^
Tertiary industry	Cubic		0.044	0.027	0.003^**^	0.015*	0.000^**^
SO_2_	Linear		0.039	0.021	0.001^**^	0.001^**^	0.000^**^
&	Quadratic	305	0.039	0.022	0.002^**^	0.235	0.000^**^
Tertiary industry	Cubic		0.046	0.021	0.003^**^	0.064	0.000^**^
NO_2_	Linear		0.120	0.012	0.000^**^	0.000^**^	0.000^**^
&	Quadratic		0.135	0.012	0.000^**^	0.000^**^	0.000^**^
Tertiary industry	Cubic		0.136	0.012	0.000^**^	0.225	0.000^**^

* P<0.05; ** P<0.01.

### 2. Variation of efficiency ratio in recent years

The ratio of the air pollutant concentration to GRP per capita (cPM_10_/GRP per capita, cSO_2_/GRP per capita and cNO_2_/GRP per capita) had a steady declining trend in the four economic regions, especially in the western mainland China, showing a notable enhancement of energy efficiency (Fig. 2). The cPM_10_/GRP per capita ratio fell from 0.103 mg m^−3^ (ten thousand yuan)^−1^ in 2003 to 0.018 in the year of 2012 by 470% in the West, from 0.064 to 0.015 in the Northeast by 320%, from 0.081 to 0.014 in the Central by 470%, and from 0.043 to 0.009 mg m^−3^ (ten thousand yuan)^−1^ in the East by 370%. The ratios of cSO_2_/GRP per capita and cNO_2_/GRP per capita also showed analogous disparity among the four regions, with the variation range of efficiency ratio the smallest in the East (Fig. 2).

### 3. EKC analysis in all the provincial capital cities and the four economic regions

**Figure 3:**
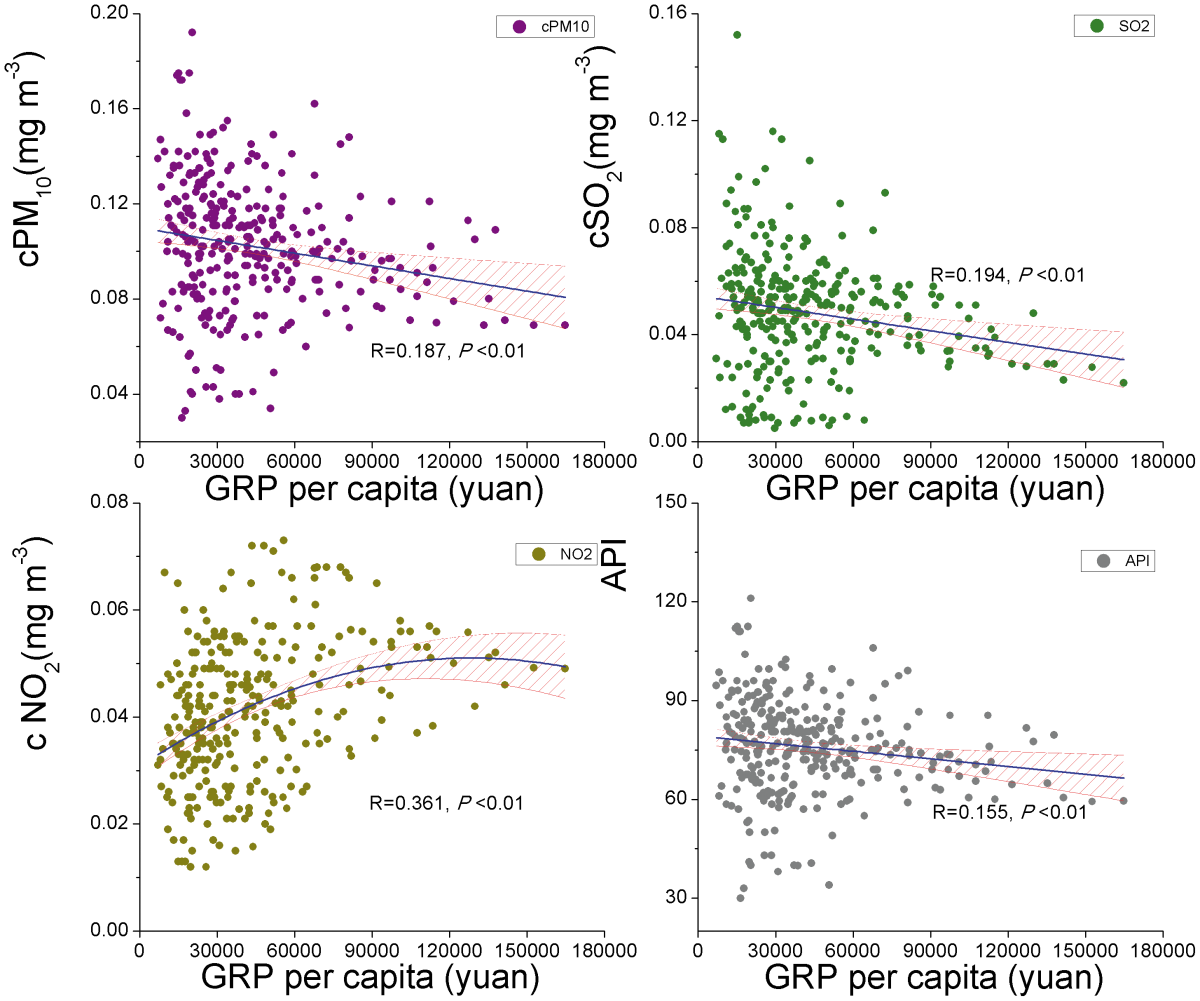
Regression curves between GRP per capita and air pollutant index (PM_10_, SO_2_, NO_2_, API) in all the provincial capital cities during 2003–2012. The blue line is the regression line and the pink area the 95% confidence limits.

The relationship between air pollutants and GRP per capita in all the provincial capitals is presented in Fig. 3. The concentration of PM_10_ and SO_2_ or API had a significantly negative linear relationship with GRP per capita; meanwhile the concentration of NO_2_ had a quadratic relationship with GRP per capita. However, the relationship between air pollutants and GRP per capita was not the same for the four economic regions (Table 2, 3). The PM_10_ concentration was significantly related to GRP per capita only in the Central. Similarly, API was also significantly related to GRP per capita only in the Central. The SO_2_ concentration had significant negative linear relationship with GRP per capita in the Central but positive linear relationship in the Northeast. The NO_2_ concentration was positively related to the GRP per capita in the Central, West, and quadratic for the East region, but not significantly related to that in the Northeast.

**Table 2:** Regression for concentration of PM_10_, SO_2_, NO_2_, API and GRP per capita (panel data of all provincial cities).

		Model summary						
	Described	Regression					Coefficients T test	
Region	Relationship	Model	n	R	SE	Sig.	Independent variable	Constant
	PM10	Linear		0.187	0.027	0.001**	0.001**	0.000**
	&	Quadratic	305	0.187	0.027	0.005**	0.267	0.000**
	GDP per capita	Cubic		0.204	0.027	0.005**	0.076	0.000**
	SO2	Linear		0.194	0.021	0.001**	0.001**	0.000**
	&	Quadratic	305	0.194	0.022	0.003**	0.439	0.000**
All	GDP per capita	Cubic		0.213	0.021	0.003**	0.084	0.000**
Provincial								
City	NO2	Linear		0.344	0.012	0.000**	0.000**	0.000**
	&	Quadratic	305	0.361	0.012	0.000**	0.000**	0.000**
	GDP per capita	Cubic		0.363	0.012	0.000**	0.361	0.000**
	API	Linear		0.155	14.327	0.007**	0.007**	0.000**
	&	Quadratic	305	0.155	14.260	0.026*	0.346	0.000**
	GDP per capital	Cubic		0.179	14.277	0.021*	0.071	0.000**

* P<0.05; ** P<0.01.

**Table 3:** Regression for concentration of PM_10_, SO_2_, NO_2_, API and GRP per capita.

		Model summary						
	Described	Regression					Coefficients T test	
Region	Relationship	Model	n	R	SE	Sig.	Independent variable	Constant
	PM_10_	Linear		0.078	0.030	0.441		
	&	Quadratic	100	0.192	0.030	0.163		
	GDP per capita	Cubic		0.192	0.030	0.305		
	SO_2_	Linear		0.096	0.022	0.343		
	&	Quadratic	100	0.228	0.022	0.076		
East	GDP per capita	Cubic		0.228	0.022	0.161		
	NO_2_	Linear		0.383	0.014	0.000^**^	0.000^**^	0.000^**^
	&	Quadratic	100	0.497	0.013	0.000^**^	0.000^**^	0.000^**^
	GDP per capita	Cubic		0.495	0.013	0.000^**^	0.016*	0.164
	API	Linear		0.283	6.517	0.129		
	&	Quadratic	100	0.285	6.633	0.318		
	GDP per capita	Cubic		0.296	6.737	0.490		
								
	PM_10_	Linear		0.449	0.018	0.000^**^	0.000^**^	0.000^**^
	&	Quadratic	60	0.465	0.018	0.001^**^	0.050*	0.000^**^
	GDP per capita	Cubic		0.474	0.018	0.002^**^	0.870	0.000^**^
	SO_2_	Linear		0.297	0.019	0.021*	0.021*	0.000^**^
	&	Quadratic	60	0.332	0.019	0.036*	0.063	0.000^**^
Central	GDP per capita	Cubic		0.391	0.019	0.082		
	NO_2_	Linear		0.386	0.010	0.002^**^	0.002^**^	0.000^**^
	&	Quadratic	60	0.386	0.010	0.010^**^	0.464	0.000^**^
	GDP per capita	Cubic		0.405	0.010	0.018*	0.244	0.116
	API	Linear		0.449	9.204	0.000^**^	0.000^**^	0.000^**^
	&	Quadratic	60	0.465	9.917	0.001^**^	0.050*	0.000^**^
	GDP per capita	Cubic		0.474	9.230	0.002^**^	0.871	0.000^**^
								
	PM_10_	Linear		0.283	0.013	0.129		
	&	Quadratic	30	0.285	0.013	0.318		
	GDP per capita	Cubic		0.296	0.013	0.490		
	SO_2_	Linear		0.463	0.012	0.010^**^	0.010^**^	0.000^**^
	&	Quadratic	30	0.463	0.013	0.038*	0.642	0.013*
Northeast	GDP per capita	Cubic		0.465	0.013	0.091		
	NO_2_	Linear		0.333	0.009	0.072		
	&	Quadratic	30	0.334	0.010	0.202		
	GDP per capita	Cubic		0.348	0.010	0.332		
	API	Linear		0.283	6.517	0.129		
	&	Quadratic	30	0.285	6.633	0.318		
	GDP per capita	Cubic		0.296	6.737	0.490		
								
	PM_10_	Linear		0.149	0.030	0.113		
	&	Quadratic	115	0.161	0.031	0.230		
	GDP per capita	Cubic		0.184	0.031	0.227		
	SO_2_	Linear		0.169	0.023	0.071		
	&	Quadratic	115	0.222	0.022	0.059		
West	GDP per capita	Cubic		0.225	0.022	0.122		
	NO_2_	Linear		0.211	0.012	0.024*	0.024*	0.000^**^
	&	Quadratic	115	0.233	0.012	0.044*	0.087	0.000^**^
	GDP per capita	Cubic		0.251	0.012	0.065		
	API	Linear		0.152	15.321	0.104		
	&	Quadratic	115	0.166	15.354	0.208		
	GDP per capita	Cubic		0.190	15.356	0.250		

Only significant P-values of T test are listed.

* P<0.05; ** P<0.01.

## Discussion

### 1. The relationship between the three main industries and air pollutants

Our results (Fig. 1) showed the quadratic relationship between the secondary and tertiary industries and NO_2_ in the provincial capital cities of China. The increase of NO_2_ concentration was probably caused by the continuous increase of civil vehicles (The civil vehicles number increased from 1.36 million to 78.0 million according to the National Bureau of Statistics) and the widespread use of transportation in many fields such as tourism. This was in agreement with the point of view ascribing anthropogenic pollution to combustion of fossil fuel [43]–[44]. Fortunately, the concentration of NO_2_ began to descend as the output of secondary and tertiary industries came to a certain level, probably due to the increasing energy efficiency (Fig. 2) and environmental-friendly measurements such as transportation control during traffic peaks [45]. Different from NO_2_, the concentration of PM_10_ and SO_2_ decreased with the increase of the industry output per capita, which could also be explained by the improved energy efficiency. The negative relationship between the output per capita of tertiary industry and PM_10_ and SO_2_ might, to a large part, attributable to the rapid development of low energy-consumption industries such as high-tech industry, though this explanation needed further confirmation. Besides, unadvanced managements such as straw burning were restricted in suburbs with the improved living standard in cities [46], which also helped to decrease the concentration of PM_10_ and SO_2_.

### 2. Variation of energy efficiency in Chinese cities

The results showed that pollutant emissions at every ten thousand yuan fell with sustainable growth of GRP in the provincial capital cities from 2003 to 2012 (Fig. 2), which was coincident with the improvement in energy and technology in Chinese industries [47]–[48]. Besides, there was a distinct difference between the high income regions and less developed ones: the more developed cities had lower concentration of air pollutants with smaller variation ranges than the less developed cities. This was probably because of the lower energy intensity and more advanced technology [49]–[50] as well as better-implemented environment-friendly policies [51] in developed cities like Beijing.

### 3. The EKC in China

The relationship curves of social economy and some air pollution indicators in a period of time present different shape at different stages of development level of the country or state [52]. SO_2_ per capita, for instance, seemed to tail off in 12 selected European countries when GRP per capita reached around 10000 dollars. Consequently, the relationship curve of SO_2_ per capita and GRP per capita appeared a declining trend [53]. Besides, the relationship curve of one air pollutant varied from another for its particular features. Carbon emissions like CO_2_ was found to increase at ever-decreasing rates, with predicted peaks beyond reasonable income level because of its cross-border externalities which result in no sufficient incentives to urge countries to regulate emission [18], [54]–[55]. These findings remind us to view the relationship of economy and air pollution with consideration of the time period and specific air pollutants [52], [56]–[57].

In the national scale, our results showed a negative relationship between GRP per capita and PM_10_, SO_2_ or API while inverted-U shape relationship with NO_2_ (Fig. 3). The EKC did exist for Chinese cities because the concentration of PM_10_ and SO_2_ stop rising from mid-1990s [58]. However, the turning point of EKC for air pollutants seemed to vary with the place, or, with the economical level. Taking SO_2_ as an example, the turning point of EKC was approximately 20 thousand yuan in Changsha-Zhuzhou-Xiangtan Urban Agglomeration [59], but 37 thousand in Beijing [60]. Peng & Bao [61] reported a national EKC knee point of around 36 thousand yuan, close to that of 30 thousand yuan claimed by Li et al [62]. Though we lacked data about SO_2_ concentration before 2003, our analysis made an estimated turning point of less than 30 thousand yuan, also consistent with other results.

**Figure 4:**
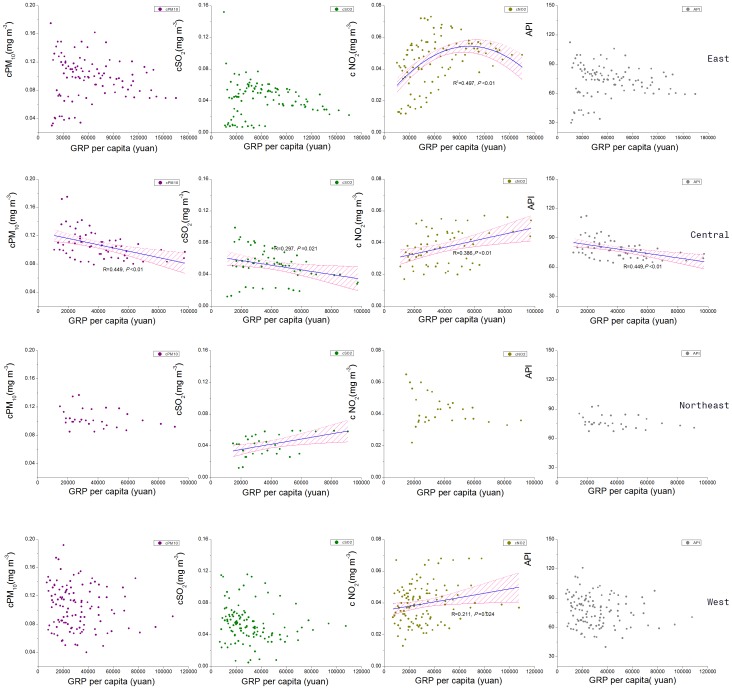
Regression curves between GRP per capita and air pollutant index (PM_10_, SO_2_, NO_2_, API) in four economic regions during 2003–2012. The blue line is the regression line and the pink area the 95% confidence limits.

Such EKC pattern was probably caused by the following: (1) The structure of Chinese economy has changed from energy-intensive heavy industry to a more market-oriented service-based economy [62], which, with its lower environmental damage [25], helped China in ameliorating the environment rather than aggravating pollution. Furthermore, in order to stay competitive, firms are keen on investing new and improved technology to enhance cost effectiveness. One of the most significant consequences of this trend is an improvement in resource use efficiency within industrial sector which cut the industrial energy intensity by 50 percent during 1990s [62]. (2) Citizens' environmental awareness is improved. As Chinese people get richer and more educated, they become more concerned about the ambient environment they dwell [63]–[65]]. At this time, their behaviors to protect environment and striving for more governmental support to do so contribute to the emergence of EKC [64], [66]. For example, a gigantic demonstration against production of p-xylene in Dalian on August 14th, 2011 [67] reflected the strong demand for better living environment. (3) Regulatory policy for environment protection has been established and effectively implemented, which is another important factor to spur EKC [68]-[69]. In China, the first law against air pollution took into effect in 1987 and was amended in 1995 and 2000. The environment-friendly measures conducted by the government also provide significant support to air quality. The central government, for example, adopted drastic new pollution control measures for town and village industrial enterprises (TVIEs) and closed 65,000 high-pollution TVIEs in a national campaign in 1996. Therefore, with strict and effective regulatory measures, air pollutants such as SO_2_, soot and industrial fugitive dust began to decrease since mid-1990s [57]. It's believed that the environment will continue to improve with Chinese central government policies making efforts to promote ecological progress [70]. However, the rise of the SO_2_ concentration in the Northeastern China simultaneous with the enhancement of civil revenues in this area (Fig. 4) might be a result of the policy of “revitalizing the old Northeast industry” by the Central Government [71].

The emergence of the knee point of EKC in the developed cities of Eastern China may be a result of well-implemented environmental policies and high investment in pollution control. But in the meantime, the Central and Northeast regions did not show a downward trend of NO_2_ concentration (Fig. 4), which was probably attributable to the growing impact of vehicular emissions [51], [72]. NO_2_ is one of the dominant components in vehicle exhaust [73]. The ever-increasing civil vehicles, particularly the surge of vehicles in the cities after 2000, probably emit enough NO_2_ to compensate the decrease of the pollutant from technical advancement of the industries [74]. It would be difficult to decrease NO_2_ concentration in most cities if civil vehicles continue to increase in the near future, despite the controlling measures already taken [75].

Since API is a simple and generalized indicator, its variation can reflect the general trend of air pollutants. The negative linear trend of API and GRP per capita in the Central region (Fig. 4) was probably attributable to the overall decreasing trend of the three categories of air pollutants (Fig. 3) [76].

It is worth noticing air pollutants were not significantly related to GRP per capita in any of the four economic regions. Since the classification criterion of the four economic regions was not only the economic development level but also including geographical location, variance of economic levels within a region might have obscured the relationship between air pollutants and GRP per capita. Some detailed classification is needed to improve the accuracy of analysis.

**Figure 5:**
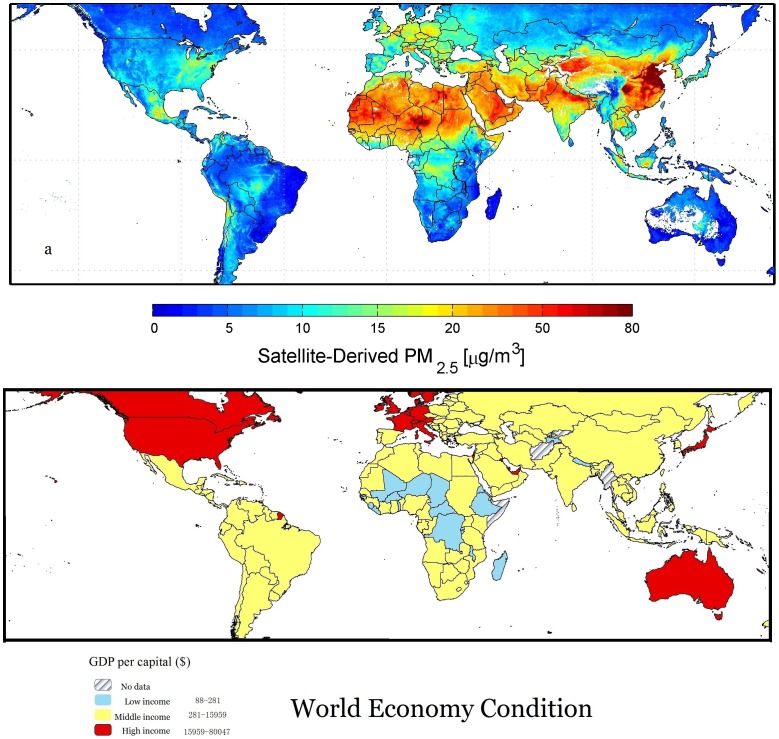
Maps of world PM_2.5_ (µg m^−3^) and GRP per capita ($) during 2001 to 2006. (a) PM_2.5_, downloaded from NASA website and reproduced with permission from its authors and publisher (van Donkelaar et al., 2010); (b) GRP per capital, derived from the World Development Indicators of the World Bank (http://data.worldbank.org/country).

Comparative qualitative analysis of the world also illustrated the existence of EKC and pointed out the developmental status of China in the world scale (Fig. 5 and Table 4). Two comparative “World Map” in Fig. 5 identified the existence of EKC at the global level. Emerging economics like China and India have the highest concentration of PM_2.5_ (Fig. 5) and PM_10_ (Table 4), mainly resulting from fossil fuel and biomass burning in order to meet the energy demand for rapid economic growth [7]. Developed countries in the Europe, Oceania and North America, however, have lower concentration of particulate matter, probably because of the following two reasons: (1) The developed countries have already accomplished the transition of industrialization which is currently taking place in developing countries like China [77]. (2) The developed countries possess more environment-friendly technology to enhance energy efficiency and reduce pollution [78], [79]. As for the undeveloped countries, most of them in Africa, the high concentration of pollutants are mainly caused by the Sahara Desert, which brings them seasonal dry, dust-laden wind known as Harmattan [80]. Of course there are other undeveloped countries with low level of pollutants, simply because they are still at the very early stage of economic development.

**Table 4:** Concentration of PM_10_ in cities of different continents.

Continent	Country(time period)	Mean PM_10_ concentration (mg m^−3^)	Scale	Reference
Asia	China(2003–2010)	0.1056±0.0259	National	This study
	Japan (2007–2008)	0.0151±0.0078	Yokohama	[81]
	India (1998–1999,2002)	0.2317±0.0815	New Delhi	[82]
Africa	South Africa (winter of 1997)	0.0933±0.0188	National	[83]
	Tanzania (2005)	0.0510±0.0210	National	[84]
	Guinea (2004)	0.1453±0.1092	Conakry	[85]
South America	Brazil (2008)	0.064±0.0190	São Paulo	[86]
	Argentina (2008)	0.0470±0.0120	Buenos Aires	
	Columbia (2008)	0.0640±0.0490	Bogotá	
Europe	(1992–2009)	0.0306±0.0084	Continental	[87]
	Netherlands (1985–2008)	0.0180	Rotterdam	[88]
	Greece (1999–2000)	0.0755±0.0275	Athens,	[89]
	German (2002–2005)	0.0663±0.0105	National	[90]
North America	US (1992–2009)	0.0276±0.0081	National	[87]
	Canada (1993–2009)	0.0155±0.0052	National	
Oceania	Australia (1998–2001)	0.0175±0.0018	National	[91]
	New Zealand (1999–2007)	0.0299±0.0132	National	[92]

## Conclusions

The quadratic relationship between the concentration of NO_2_ and the output per capita of the secondary or tertiary industry, as well as the negative correlation between the concentration of PM_10_, SO_2_ and industry output per capita, indicate the declining trend of the pollutant concentrations with the improvement of energy efficiency and implementation of environment protection policies. With technology innovation and modulation in industries together with policy implementation from 1990s, ratios of pollutants to GRP in the 31 provincial capitals in mainland China shows a downward trend. Such negative or invert-U quadratic relationship curve between air pollutants and GRP per capita verifies the existence of EKC in China.

## Supporting Information

Figure S1 **A hazy day (a: January 29, 2013) and a fine day (b: February 1, 2013) in downtown Beijing.** (Pictures from http://ndphotos.oeeee.com/album/201302/01/2140.html?id=1).
(TIF)

Table S1 Emission inventories of provincial cities in mainland China.
(DOC)

## References

[1] Davis DL (2002) A look back at the London smog of 1952 and the half century since. Environmental health perspectives 110: A734. 12501843 10.1289/ehp.110-a734 PMC1241116

[2] Akimoto H (2003) Global air quality and pollution. Science 302: 1716–1719. 14657488 10.1126/science.1092666

[3] Kopp RE, Mauzerall DL (2010) Assessing the climatic benefits of black carbon mitigation. Proc Natl Acad Sci U S A 107: 11703–11708. 20566891 10.1073/pnas.0909605107 PMC2900637

[4] Ramanathan V, Feng Y (2009) Air pollution, greenhouse gases and climate change: Global and regional perspectives. Atmospheric Environment 43: 37–50.

[5] Clarke A, Kapustin V (2010) Hemispheric Aerosol Vertical Profiles: Anthropogenic Impacts on Optical Depth and Cloud Nuclei. Science 329: 1488–1492. 20847262 10.1126/science.1188838

[6] Ramanathan V, Chung C, Kim D, Bettge T, Buja L, et al (2005) Atmospheric brown clouds: Impacts on South Asian climate and hydrological cycle. Proc Natl Acad Sci U S A 102: 5326–5333. 15749818 10.1073/pnas.0500656102 PMC552786

[7] Allen RJ, Sherwood SC, Norris JR, Zender CS (2012) Recent Northern Hemisphere tropical expansion primarily driven by black carbon and tropospheric ozone. Nature 485: 350–U393. 22596159 10.1038/nature11097

[8] Bollasina MA, Ming Y, Ramaswamy V (2011) Anthropogenic aerosols and the weakening of the South Asian summer monsoon. Science 334: 502–505. 21960529 10.1126/science.1204994

[9] Booth BB, Dunstone NJ, Halloran PR, Andrews T, Bellouin N (2012) Aerosols implicated as a prime driver of twentieth-century North Atlantic climate variability. Nature 484: 228–232. 22498628 10.1038/nature10946

[10] Menon S, Hansen J, Nazarenko L, Luo YF (2002) Climate effects of black carbon aerosols in China and India. Science 297: 2250–2253. 12351786 10.1126/science.1075159

[11] Silva RA, West JJ, Zhang Y, Anenberg SC, Lamarque J-F, et al (2013) Global premature mortality due to anthropogenic outdoor air pollution and the contribution of past climate change. Environmental Research Letters 8: 034005.

[12] Dadvand P, de Nazelle A, Triguero-Mas M, Schembari A, Cirach M, et al (2012) Surrounding greenness and exposure to air pollution during pregnancy: an analysis of personal monitoring data. Environmental health perspectives 120: 1286. 22647671 10.1289/ehp.1104609 PMC3440116

[13] Tonne C, Wilkinson P (2013) Long-term exposure to air pollution is associated with survival following acute coronary syndrome. European heart journal 34: 1306–1311. 23423735 10.1093/eurheartj/ehs480 PMC3640199

[14] Chen Y, Ebenstein A, Greenstone M, Li H (2013) Evidence on the impact of sustained exposure to air pollution on life expectancy from China's Huai River policy. Proceedings of the National Academy of Sciences. 10.1073/pnas.1300018110 PMC3740827 23836630

[15] Crandall RW (1983) Controlling industrial pollution: The economics and politics of clean air.

[16] Seaton A, Godden D, MacNee W, Donaldson K (1995) Particulate air pollution and acute health effects. The Lancet 345: 176–178. 10.1016/s0140-6736(95)90173-6 7741860

[17] Stieb DM, Judek S, Burnett RT (2002) Meta-analysis of time-series studies of air pollution and mortality: effects of gases and particles and the influence of cause of death, age, and season. Journal of the Air & Waste Management Association 52: 470–484. 12002192 10.1080/10473289.2002.10470794

[18] Galeotti M (2003) Economic Development and Environmental Protection. Fondazione Eni Enrico Mattei 2003: 89.

[19] Carson RT (2010) The environmental Kuznets curve: seeking empirical regularity and theoretical structure. Review of Environmental Economics and Policy 4: 3–23.

[20] Selden TM, Song D (1994) Environmental quality and development: is there a Kuznets curve for air pollution emissions? Journal of Environmental Economics and management 27: 147–162.

[21] Shafik N (1994) Economic development and environmental quality: an econometric analysis. Oxford Economic Papers: 757–773.

[22] Cole MA, Rayner AJ, Bates JM (1997) The environmental Kuznets curve: an empirical analysis. Environment and development economics 2: 401–416.

[23] De Bruyn SM, van den Bergh JC, Opschoor JB (1998) Economic growth and emissions: reconsidering the empirical basis of environmental Kuznets curves. Ecological Economics 25: 161–175.

[24] Grossman GM, Krueger AB (1991) Environmental impacts of a North American free trade agreement. National Bureau of Economic Research.

[25] Panayotou T (1993) Empirical tests and policy analysis of environmental degradation at different stages of economic development. International Labour Organization.

[26] Wang Y-C (2011) Short-and Long-run Environmental Kuznets Curve: Case Studies of Sulfur Emissions in OECD Countries International Journal of Economic Research. 9: 1–18.

[27] Akbostancı E, Türüt-Aşık S, Tunç Gİ (2009) The relationship between income and environment in Turkey: Is there an environmental Kuznets curve? Energy Policy 37: 861–867.

[28] Fodha M, Zaghdoud O (2010) Economic growth and pollutant emissions in Tunisia: An empirical analysis of the environmental Kuznets curve. Energy Policy 38: 1150–1156.

[29] De Groot HL, Withagen CA, Minliang Z (2004) Dynamics of China's regional development and pollution: an investigation into the Environmental Kuznets Curve. Environment and development economics 9: 507–537.

[30] Jalil A, Mahmud SF (2009) Environment Kuznets curve for CO_2_emissions: A cointegration analysis for China. Energy Policy 37: 5167–5172.

[31] Vincent JR (1997) Testing for environmental Kuznets curves within a developing country. Environment and development economics 2: 417–431.

[32] Song T, Zheng T, Tong L (2008) An empirical test of the environmental Kuznets curve in China: A panel cointegration approach. China Economic Review 19: 381–392.

[33] Ren W-X, Xue B, Zhang L, Ma Z-X, Geng Y (2013) Spatiotemporal variations of air pollution index in China's megacities. Chinese Journal of Ecology 32 (10):2788–2796, in Chinese with english abstract.

[34] GAO B-L (2009) Relationship Between Environmental Pollution and Economic Growth in Jiangsu Province. POLLUTION CONTROL TECHNOLOGY 22(6):36–39, in Chinese with english abstract.

[35] Li H-Y, Li J, Zhang D-H, Chen S-S (2013) The Environmental Kuznets Curve in Xi'an. ARID ZONE RESEARCH 30(3): 556–562, in Chinese with english abstract.

[36] Shen J (2006) A simultaneous estimation of Environmental Kuznets Curve: Evidence from China. China Economic Review 17: 383–394.

[37] Hu M-X, Hu H, Liu Z (2005) Air Environmental Kuznets Curve (EKC) Characteristics in Wuhan City. Journal of Wuhan Polytechnic University 24(1):72–75, in Chinese with english abstract.

[38] He K, Huo H, Zhang Q (2002) Urban air pollution in China: current status, characteristics, and progress. Annual Review of Energy and the Environment 27: 397–431.

[39] Ministry of Environmental Protection of the People's Republic of China (2012) http://kjs.mep.gov.cn/hjbhbz/bzwb/dqhjbh/jcgfffbz/201203/t20120302_224166.htm.

[40] Wikipedia. (2013). http://en.wikipedia.org/wiki/List_of_regions_of_China.

[41] Agras J, Chapman D (1999) A dynamic approach to the Environmental Kuznets Curve hypothesis. Ecological Economics 28: 267–277.

[42] LI R-e, ZHANG H-j (2008) An empirical analysis on the regional discrepancy and tendency of EKC in China (1981–2004). Journal of Xi'an Jiaotong University (Social Sciences) 4: 007.

[43] Kampa M, Castanas E (2008) Human health effects of air pollution. Environmental Pollution 151: 362–367. 17646040 10.1016/j.envpol.2007.06.012

[44] Gustafsson Ö, Kruså M, Zencak Z, Sheesley RJ, Granat L, et al (2009) Brown clouds over South Asia: biomass or fossil fuel combustion? Science 323: 495–498. 19164746 10.1126/science.1164857

[45] Zhou Y, Wu Y, Yang L, Fu L, He K, et al (2010) The impact of transportation control measures on emission reductions during the 2008 Olympic Games in Beijing, China. Atmospheric Environment 44: 285–293.

[46] Kim Oanh NT, Ly BT, Tipayarom D, Manandhar BR, Prapat P, et al (2011) Characterization of particulate matter emission from open burning of rice straw. Atmospheric Environment 45: 493–502. 21243095 10.1016/j.atmosenv.2010.09.023 PMC3018782

[47] Sinton JE, Fridley DG (2000) What goes up: recent trends in China's energy consumption. Energy Policy 28: 671–687.

[48] Hu J-L, Wang S-C (2006) Total-factor energy efficiency of regions in China. Energy Policy 34: 3206–3217. 10.1016/j.enpol.2013.07.124 PMC7115794 32287870

[49] Dhakal S (2009) Urban energy use and carbon emissions from cities in China and policy implications. Energy Policy 37: 4208–4219.

[50] Fan J (2006) Industrial Agglomeration and Difference of Regional Labor Productivity: Chinese Evidence with International Comparison [J]. Economic Research Journal 11: 72–81.

[51] Li L, Chen C, Xie S, Huang C, Cheng Z, et al (2010) Energy demand and carbon emissions under different development scenarios for Shanghai, China. Energy Policy 38: 4797–4807.

[52] Levinson A (2002) The ups and downs of the environmental Kuznets curve. Recent Advances in Environmental Economics Edward Elgar, Cheltenham: 119–139.

[53] Markandya A, Golub A, Pedroso-Galinato S (2006) Empirical analysis of national income and SO_2_ emissions in selected European countries. Environmental and resource economics 35: 221–257.

[54] Lieb CM (2004) The environmental Kuznets curve and flow versus stock pollution: the neglect of future damages. Environmental and resource economics 29: 483–506.

[55] Miah D, Masum FH (2010) Global observation of EKC hypothesis for CO_2_, SO_2_ and NO_2_ emission: A policy understanding for climate change mitigation in Bangladesh. Energy Policy 38: 4643–4651.

[56] Figueroa B, Pastén C (2009) Country specific environmental Kuznets curves: A random coefficient approach applied to high-income countries. Estudios de Economía 36: 5–32.

[57] Harris PG (2006) Environmental Perspectives and Behavior in China Synopsis and Bibliography. Environment and Behavior 38: 5–21.

[58] Zeng H-X, Qin D-L, Luo F, Zhang H, Luo Y-P (2010) Study on the Relationship between Enironmetal Pollution and Economic Growth in the Changsha - Zhuzhou - Xiangtan Urban Agglomera tion. Ecological Economy 1:347–350, in Chinese with english abstract.

[59] Zhou Y-M, Huang S-P (2010) Research on Relationship between Economic Growth and Environmental Pollution: an Empirical Analysis Based on Panel Data of Beijing. in Chinese with english abstract.

[60] Peng S-J, Bao Q (2006) Economic Growth and Environmental Pollution: An Empirical Test for the Environmental Kuznets Curve Hypothesis in China. Research on Financial and Economic Issues 8: 3–17.

[61] Li R-P, Wang G-S, Wang A-J, Luo J-H, Geng N (2010). Factor Analysis of SO2 Emission Trend in Typical Industrialized Countries and Its Revelation to China. Acta Geoscientica Sinica 31(5): 749–758, in Chinese with english abstract.

[62] World Bank (2001) China: air, land, and water: environmental priorities for a new millennium. Washington, DC: World Bank.

[63] Liu J, Diamond J (2005) China's environment in a globalizing world. Nature 435: 1179–1186. 15988514 10.1038/4351179a

[64] Guo X, Marinova D (2011) Environmental awareness in China: Facilitating the greening of the economy.

[65] Martens 1 S (2006) Public participation with Chinese characteristics: citizen consumers in China's environmental management. Environmental politics 15: 211–230.

[66] Xie L (2011) China's environmental activism in the age of globalization. Asian Politics & Policy 3: 207–224.

[67] BBC News. (2011) China protest closes toxic chemical plant in Dalian. http://www.bbc.co.uk/news/world-asia-pacific-14520438

[68] He J, Wang H (2012) Economic structure, development policy and environmental quality: An empirical analysis of environmental Kuznets curves with Chinese municipal data. Ecological Economics 76: 49–59.

[69] Kijima M, Nishide K, Ohyama A (2011) EKC-type transitions and environmental policy under pollutant uncertainty and cost irreversibility. Journal of Economic Dynamics and Control 35: 746–763.

[70] Xinhua News Agency. (2012) Full text of Hu Jintao's report at 18th Party Congress. http://news.xinhuanet.com/english/special/18cpcnc/2012-11/17/c_131981259.htm

[71] Zhang P, Ma Y, Liu W, Chen Q (2004) New Urbanization Strategy for Revitalizing the Tradianal Industrial Base of Northeast China. Acta Geographica Sinica: S1.

[72] World Bank. (1997). Clear Water, blue skies: China's environmental pollution in the new century. Washington, DC: World Bank.

[73] Brugge D, Durant JL, Rioux C (2007) Near-highway pollutants in motor vehicle exhaust: a review of epidemiologic evidence of cardiac and pulmonary health risks. Environmental Health 6: 23. 17688699 10.1186/1476-069X-6-23 PMC1971259

[74] National Bureau of Statistics. (2011) http://www.stats.gov.cn/tjsj/ndsj/2011/indexch.htm.

[75] Li X-F, Zhang M-J, Wang S-J, Zhao A-F, Ma Q (2012) Variation Characteristics and Influencing Factors of Air Pollution Index in China. Environmental Science 33(6): 1936-1943, in Chinese with english abstract. 22946179

[76] Wang L, Jang C, Zhang Y, Wang K, Zhang Q, et al (2010) Assessment of air quality benefits from national air pollution control policies in China. Part II: Evaluation of air quality predictions and air quality benefits assessment. Atmospheric Environment 44: 3449–3457.

[77] Great Lakes Invitational Conference Association (GLICA). (2008) Promoting Sustainable Industry in the Developing World. http://www.glica.org/topics/show/53

[78] Dechezleprêtre A, Glachant M, Haščič I, Johnstone N, Ménière Y (2011) Invention and transfer of climate change–mitigation technologies: a global analysis. Review of Environmental Economics and Policy 5: 109–130.

[79] Popp D (2011) International technology transfer, climate change, and the clean development mechanism. Review of Environmental Economics and Policy 5: 131–152.

[80] Afeti G, Resch F (2000) Physical characteristics of Saharan dust near the Gulf of Guinea. Atmospheric Environment 34: 1273–1279.

[81] Khan MF, Shirasuna Y, Hirano K, Masunaga S (2010) Characterization of PM2.5, PM2.5–10 and PM> 10 in ambient air, Yokohama, Japan. Atmospheric Research 96: 159–172.

[82] Monkkonen P, Uma R, Srinivasan D, Koponen IK, Lehtinen KEJ, et al (2004) Relationship and variations of aerosol number and PM10 mass concentrations in a highly polluted urban environment - New Delhi, India. Atmospheric Environment 38: 425–433.

[83] Engelbrecht JP, Swanepoel L, Chow JC, Watson JG, Egami RT (2001) PM2.5 and PM10 concentrations from the Qalabotjha low-smoke fuels macro-scale experiment in South Africa. Environmental Monitoring and Assessment 69: 1–15. 11393541 10.1023/a:1010786615180

[84] Mkoma SL, Maenhaut W, Chi XG, Wang W, Raes N (2009) Characterisation of PM10 atmospheric aerosols for the wet season 2005 at two sites in East Africa. Atmospheric Environment 43: 631–639.

[85] Weinstein JP, Hedges SR, Kimbrough S (2010) Characterization and aerosol mass balance of PM2.5 and PM10 collected in Conakry, Guinea during the 2004 Harmattan period. Chemosphere 78: 980–988. 20045175 10.1016/j.chemosphere.2009.12.022

[86] Vasconcellos PC, Souza DZ, Avila SG, Araujo MP, Naoto E, et al (2011) Comparative study of the atmospheric chemical composition of three South American cities. Atmospheric Environment 45: 5770–5777.

[87] Wang KC, Dickinson RE, Su L, Trenberth KE (2012) Contrasting trends of mass and optical properties of aerosols over the Northern Hemisphere from 1992 to 2011. Atmospheric Chemistry and Physics 12: 9387–9398.

[88] Keuken M, Zandveld P, van den Elshout S, Janssen NAH, Hoek G (2011) Air quality and health impact of PM10 and EC in the city of Rotterdam, the Netherlands in 1985-2008. Atmospheric Environment 45: 5294–5301.

[89] Chaloulakou A, Kassomenos P, Spyrellis N, Demokritou P, Koutrakis P (2003) Measurements of PM10 and PM2.5 particle concentrations in Athens, Greece. Atmospheric Environment 37: 649–660.

[90] Buns C, Klemm O, Wurzler S, Hebbinghaus H, Steckelbach I, et al (2012) Comparison of four years of air pollution data with a mesoscale model. Atmospheric Research 118: 404–417.

[91] Barnett AG, Williams GM, Schwartz J, Neller AH, Best TL, et al (2005) Air pollution and child respiratory health - A case-crossover study in Australia and new Zealand. American Journal of Respiratory and Critical Care Medicine 171: 1272–1278. 15764722 10.1164/rccm.200411-1586OC

[92] Trompetter WJ, Davy PK, Markwitz A (2010) Influence of environmental conditions on carbonaceous particle concentrations within New Zealand. Journal of Aerosol Science 41: 134–142.

