# Supplementary figures and images for: Relationship between Air Pollutants and Economic Development of the Provincial Capital Cities in China during the Past Decade
Source: PLoS One. 2014 Aug 1;9(8):e104013. doi: 10.1371/journal.pone.0104013 (PMC4119013; doi:10.1371/journal.pone.0104013)

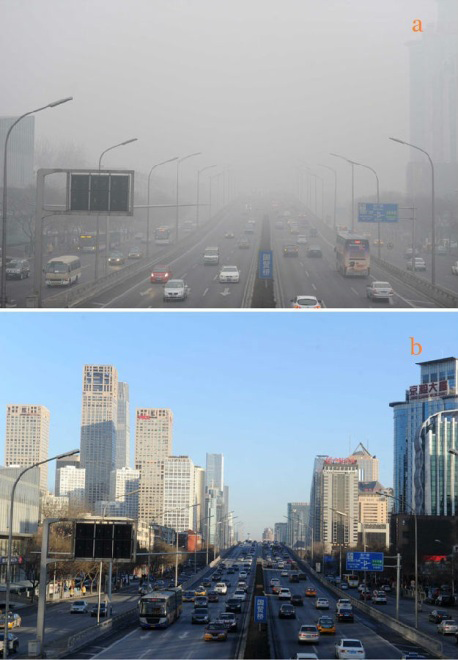

Supplement: Figure S1 — A hazy day (a: January 29, 2013) and a fine day (b: February 1, 2013) in downtown Beijing. (Pictures from http://ndphotos.oeeee.com/album/201302/01/2140.html?id=1). (TIF) [file pone.0104013.s001.tif]
