# Supplementary material for: Relationship between Air Pollutants and Economic Development of the Provincial Capital Cities in China during the Past Decade
Source: PLoS One. 2014 Aug 1;9(8):e104013. doi: 10.1371/journal.pone.0104013 (PMC4119013; doi:10.1371/journal.pone.0104013)
Supplement: Table S1 — Emission inventories of provincial cities in mainland China. (DOC) [file pone.0104013.s002.doc]

Table S1 Emission inventories of provincial cities in mainland China

| **City** | **Year** | **PM_10_** | **SO_2_** | **NO_2_** |
| --- | --- | --- | --- | --- |
| **Guangzhou** | 2003 | 0.10 | 0.06 | 0.07 |
| **ShangHai** | 2003 | 0.10 | 0.04 | 0.06 |
| **Beijing** | 2003 | 0.14 | 0.06 | 0.07 |
| **Tianjin** | 2003 | 0.13 | 0.07 | 0.05 |
| **Hangzhou** | 2003 | 0.12 | 0.05 | 0.06 |
| **Nanjing** | 2003 | 0.12 | 0.03 | 0.05 |
| **Jinan** | 2003 | 0.15 | 0.06 | 0.05 |
| **Fuzhou** | 2003 | 0.08 | 0.01 | 0.03 |
| **Haikou** | 2003 | 0.03 | 0.01 | 0.01 |
| **Shijiazhuang** | 2003 | 0.18 | 0.15 | 0.04 |
| **Changsha** | 2003 | 0.14 | 0.08 | 0.04 |
| **Wuhan** | 2003 | 0.13 | 0.05 | 0.05 |
| **Hefei** | 2003 | 0.10 | 0.01 | 0.03 |
| **Taiyuan** | 2003 | 0.17 | 0.10 | 0.03 |
| **Nanchang** | 2003 | 0.10 | 0.05 | 0.03 |
| **Zhengzhou** | 2003 | 0.11 | 0.05 | 0.03 |
| **Shenyang** | 2003 | 0.14 | 0.05 | 0.04 |
| **Changchun** | 2003 | 0.10 | 0.01 | 0.02 |
| **Harbin** | 2003 | 0.12 | 0.04 | 0.07 |
| **Hohhot** | 2003 | 0.12 | 0.04 | 0.05 |
| **Urumqi** | 2003 | 0.13 | 0.10 | 0.06 |
| **Yinchuan** | 2003 | 0.13 | 0.06 | 0.04 |
| **Chengdu** | 2003 | 0.12 | 0.05 | 0.05 |
| **Xi'an** | 2003 | 0.14 | 0.06 | 0.04 |
| **Kunming** | 2003 | 0.09 | 0.05 | 0.03 |
| **Lanzhou** | 2003 | 0.17 | 0.09 | 0.05 |
| **Guiyang** | 2003 | 0.10 | 0.09 | 0.02 |
| **Xining** | 2003 | 0.14 | 0.03 | 0.03 |
| **Nanning** | 2003 | 0.07 | 0.05 | 0.03 |
| **Chongqing** | 2003 | 0.15 | 0.12 | 0.05 |
| **Lhasa** | 2003 | 0.07 | 0.00 | 0.03 |
| **Guangzhou** | 2004 | 0.10 | 0.08 | 0.07 |
| **ShangHai** | 2004 | 0.10 | 0.06 | 0.06 |
| **Beijing** | 2004 | 0.15 | 0.06 | 0.07 |
| **Tianjin** | 2004 | 0.11 | 0.07 | 0.05 |
| **Hangzhou** | 2004 | 0.11 | 0.05 | 0.06 |
| **Nanjing** | 2004 | 0.12 | 0.05 | 0.06 |
| **Jinan** | 2004 | 0.15 | 0.05 | 0.04 |
| **Fuzhou** | 2004 | 0.07 | 0.01 | 0.04 |
| **Haikou** | 2004 | 0.03 | 0.01 | 0.01 |
| **Shijiazhuang** | 2004 | 0.12 | 0.09 | 0.04 |
| **Changsha** | 2004 | 0.14 | 0.08 | 0.03 |
| **Wuhan** | 2004 | 0.13 | 0.05 | 0.05 |
| **Hefei** | 2004 | 0.11 | 0.01 | 0.02 |
| **Taiyuan** | 2004 | 0.18 | 0.09 | 0.02 |
| **Nanchang** | 2004 | 0.10 | 0.06 | 0.03 |
| **Zhengzhou** | 2004 | 0.11 | 0.06 | 0.04 |
| **Shenyang** | 2004 | 0.14 | 0.05 | 0.04 |
| **Changchun** | 2004 | 0.09 | 0.01 | 0.03 |
| **Harbin** | 2004 | 0.11 | 0.04 | 0.06 |
| **Hohhot** | 2004 | 0.08 | 0.05 | 0.04 |
| **Urumqi** | 2004 | 0.11 | 0.10 | 0.06 |
| **Yinchuan** | 2004 | 0.12 | 0.05 | 0.04 |
| **Chengdu** | 2004 | 0.12 | 0.07 | 0.05 |
| **Xi'an** | 2004 | 0.14 | 0.05 | 0.03 |
| **Kunming** | 2004 | 0.09 | 0.07 | 0.04 |
| **Lanzhou** | 2004 | 0.17 | 0.07 | 0.05 |
| **Guiyang** | 2004 | 0.08 | 0.09 | 0.02 |
| **Xining** | 2004 | 0.13 | 0.02 | 0.03 |
| **Nanning** | 2004 | 0.08 | 0.06 | 0.03 |
| **Chongqing** | 2004 | 0.14 | 0.11 | 0.07 |
| **Lhasa** | 2004 | 0.05 | 0.00 | 0.02 |
| **Guangzhou** | 2005 | 0.09 | 0.05 | 0.07 |
| **ShangHai** | 2005 | 0.09 | 0.06 | 0.06 |
| **Beijing** | 2005 | 0.14 | 0.05 | 0.07 |
| **Tianjin** | 2005 | 0.11 | 0.08 | 0.05 |
| **Hangzhou** | 2005 | 0.11 | 0.06 | 0.06 |
| **Nanjing** | 2005 | 0.11 | 0.05 | 0.05 |
| **Jinan** | 2005 | 0.13 | 0.06 | 0.02 |
| **Fuzhou** | 2005 | 0.07 | 0.02 | 0.04 |
| **Haikou** | 2005 | 0.04 | 0.01 | 0.02 |
| **Shijiazhuang** | 2005 | 0.13 | 0.05 | 0.04 |
| **Changsha** | 2005 | 0.12 | 0.08 | 0.04 |
| **Wuhan** | 2005 | 0.12 | 0.05 | 0.05 |
| **Hefei** | 2005 | 0.10 | 0.02 | 0.03 |
| **Taiyuan** | 2005 | 0.14 | 0.08 | 0.02 |
| **Nanchang** | 2005 | 0.09 | 0.05 | 0.03 |
| **Zhengzhou** | 2005 | 0.11 | 0.06 | 0.04 |
| **Shenyang** | 2005 | 0.12 | 0.05 | 0.04 |
| **Changchun** | 2005 | 0.10 | 0.03 | 0.04 |
| **Harbin** | 2005 | 0.10 | 0.04 | 0.06 |
| **Hohhot** | 2005 | 0.10 | 0.05 | 0.04 |
| **Urumqi** | 2005 | 0.11 | 0.12 | 0.06 |
| **Yinchuan** | 2005 | 0.09 | 0.05 | 0.03 |
| **Chengdu** | 2005 | 0.13 | 0.08 | 0.05 |
| **Xi'an** | 2005 | 0.13 | 0.04 | 0.03 |
| **Kunming** | 2005 | 0.08 | 0.06 | 0.04 |
| **Lanzhou** | 2005 | 0.16 | 0.07 | 0.04 |
| **Guiyang** | 2005 | 0.08 | 0.06 | 0.01 |
| **Xining** | 2005 | 0.11 | 0.03 | 0.03 |
| **Nanning** | 2005 | 0.07 | 0.06 | 0.04 |
| **Chongqing** | 2005 | 0.12 | 0.07 | 0.05 |
| **Lhasa** | 2005 | 0.07 | 0.01 | 0.03 |
| **Guangzhou** | 2006 | 0.08 | 0.05 | 0.07 |
| **ShangHai** | 2006 | 0.09 | 0.05 | 0.06 |
| **Beijing** | 2006 | 0.16 | 0.05 | 0.07 |
| **Tianjin** | 2006 | 0.11 | 0.07 | 0.05 |
| **Hangzhou** | 2006 | 0.11 | 0.06 | 0.06 |
| **Nanjing** | 2006 | 0.11 | 0.06 | 0.05 |
| **Jinan** | 2006 | 0.11 | 0.04 | 0.02 |
| **Fuzhou** | 2006 | 0.07 | 0.02 | 0.05 |
| **Haikou** | 2006 | 0.04 | 0.01 | 0.01 |
| **Shijiazhuang** | 2006 | 0.14 | 0.04 | 0.04 |
| **Changsha** | 2006 | 0.11 | 0.08 | 0.04 |
| **Wuhan** | 2006 | 0.12 | 0.06 | 0.05 |
| **Hefei** | 2006 | 0.10 | 0.02 | 0.03 |
| **Taiyuan** | 2006 | 0.14 | 0.08 | 0.03 |
| **Nanchang** | 2006 | 0.09 | 0.06 | 0.03 |
| **Zhengzhou** | 2006 | 0.11 | 0.06 | 0.04 |
| **Shenyang** | 2006 | 0.12 | 0.06 | 0.04 |
| **Changchun** | 2006 | 0.10 | 0.03 | 0.04 |
| **Harbin** | 2006 | 0.10 | 0.03 | 0.05 |
| **Hohhot** | 2006 | 0.10 | 0.05 | 0.05 |
| **Urumqi** | 2006 | 0.15 | 0.11 | 0.06 |
| **Yinchuan** | 2006 | 0.10 | 0.05 | 0.03 |
| **Chengdu** | 2006 | 0.12 | 0.07 | 0.05 |
| **Xi'an** | 2006 | 0.13 | 0.06 | 0.04 |
| **Kunming** | 2006 | 0.09 | 0.06 | 0.04 |
| **Lanzhou** | 2006 | 0.19 | 0.06 | 0.05 |
| **Guiyang** | 2006 | 0.08 | 0.07 | 0.02 |
| **Xining** | 2006 | 0.14 | 0.02 | 0.03 |
| **Nanning** | 2006 | 0.07 | 0.06 | 0.04 |
| **Chongqing** | 2006 | 0.11 | 0.07 | 0.05 |
| **Lhasa** | 2006 | 0.06 | 0.01 | 0.03 |
| **Guangzhou** | 2007 | 0.08 | 0.05 | 0.07 |
| **ShangHai** | 2007 | 0.09 | 0.06 | 0.05 |
| **Beijing** | 2007 | 0.15 | 0.05 | 0.07 |
| **Tianjin** | 2007 | 0.09 | 0.06 | 0.04 |
| **Hangzhou** | 2007 | 0.11 | 0.06 | 0.06 |
| **Nanjing** | 2007 | 0.11 | 0.06 | 0.05 |
| **Jinan** | 2007 | 0.12 | 0.06 | 0.02 |
| **Fuzhou** | 2007 | 0.07 | 0.03 | 0.06 |
| **Haikou** | 2007 | 0.04 | 0.01 | 0.01 |
| **Shijiazhuang** | 2007 | 0.13 | 0.04 | 0.04 |
| **Changsha** | 2007 | 0.10 | 0.07 | 0.04 |
| **Wuhan** | 2007 | 0.12 | 0.06 | 0.06 |
| **Hefei** | 2007 | 0.12 | 0.02 | 0.03 |
| **Taiyuan** | 2007 | 0.12 | 0.08 | 0.03 |
| **Nanchang** | 2007 | 0.08 | 0.05 | 0.03 |
| **Zhengzhou** | 2007 | 0.11 | 0.07 | 0.05 |
| **Shenyang** | 2007 | 0.12 | 0.05 | 0.04 |
| **Changchun** | 2007 | 0.10 | 0.03 | 0.04 |
| **Harbin** | 2007 | 0.10 | 0.05 | 0.06 |
| **Hohhot** | 2007 | 0.08 | 0.07 | 0.05 |
| **Urumqi** | 2007 | 0.14 | 0.09 | 0.07 |
| **Yinchuan** | 2007 | 0.09 | 0.05 | 0.03 |
| **Chengdu** | 2007 | 0.11 | 0.06 | 0.05 |
| **Xi'an** | 2007 | 0.14 | 0.05 | 0.04 |
| **Kunming** | 2007 | 0.08 | 0.07 | 0.04 |
| **Lanzhou** | 2007 | 0.13 | 0.06 | 0.04 |
| **Guiyang** | 2007 | 0.09 | 0.06 | 0.02 |
| **Xining** | 2007 | 0.12 | 0.03 | 0.04 |
| **Nanning** | 2007 | 0.06 | 0.06 | 0.05 |
| **Chongqing** | 2007 | 0.11 | 0.07 | 0.04 |
| **Lhasa** | 2007 | 0.06 | 0.01 | 0.03 |
| **Guangzhou** | 2008 | 0.07 | 0.05 | 0.06 |
| **ShangHai** | 2008 | 0.08 | 0.05 | 0.06 |
| **Beijing** | 2008 | 0.12 | 0.04 | 0.05 |
| **Tianjin** | 2008 | 0.09 | 0.06 | 0.04 |
| **Hangzhou** | 2008 | 0.11 | 0.05 | 0.05 |
| **Nanjing** | 2008 | 0.10 | 0.05 | 0.05 |
| **Jinan** | 2008 | 0.13 | 0.05 | 0.02 |
| **Fuzhou** | 2008 | 0.07 | 0.02 | 0.05 |
| **Haikou** | 2008 | 0.04 | 0.01 | 0.02 |
| **Shijiazhuang** | 2008 | 0.12 | 0.05 | 0.03 |
| **Changsha** | 2008 | 0.10 | 0.05 | 0.04 |
| **Wuhan** | 2008 | 0.11 | 0.05 | 0.05 |
| **Hefei** | 2008 | 0.13 | 0.02 | 0.03 |
| **Taiyuan** | 2008 | 0.09 | 0.07 | 0.02 |
| **Nanchang** | 2008 | 0.08 | 0.05 | 0.04 |
| **Zhengzhou** | 2008 | 0.09 | 0.06 | 0.05 |
| **Shenyang** | 2008 | 0.12 | 0.06 | 0.04 |
| **Changchun** | 2008 | 0.10 | 0.03 | 0.04 |
| **Harbin** | 2008 | 0.10 | 0.04 | 0.06 |
| **Hohhot** | 2008 | 0.07 | 0.05 | 0.05 |
| **Urumqi** | 2008 | 0.15 | 0.11 | 0.07 |
| **Yinchuan** | 2008 | 0.08 | 0.05 | 0.02 |
| **Chengdu** | 2008 | 0.11 | 0.05 | 0.05 |
| **Xi'an** | 2008 | 0.11 | 0.05 | 0.04 |
| **Kunming** | 2008 | 0.07 | 0.05 | 0.04 |
| **Lanzhou** | 2008 | 0.13 | 0.07 | 0.05 |
| **Guiyang** | 2008 | 0.08 | 0.06 | 0.02 |
| **Xining** | 2008 | 0.12 | 0.03 | 0.03 |
| **Nanning** | 2008 | 0.06 | 0.04 | 0.04 |
| **Chongqing** | 2008 | 0.11 | 0.06 | 0.04 |
| **Lhasa** | 2008 | 0.05 | 0.01 | 0.02 |
| **Guangzhou** | 2009 | 0.07 | 0.04 | 0.06 |
| **ShangHai** | 2009 | 0.08 | 0.04 | 0.05 |
| **Beijing** | 2009 | 0.12 | 0.03 | 0.05 |
| **Tianjin** | 2009 | 0.10 | 0.06 | 0.04 |
| **Hangzhou** | 2009 | 0.10 | 0.04 | 0.05 |
| **Nanjing** | 2009 | 0.10 | 0.04 | 0.05 |
| **Jinan** | 2009 | 0.12 | 0.05 | 0.03 |
| **Fuzhou** | 2009 | 0.06 | 0.01 | 0.04 |
| **Haikou** | 2009 | 0.04 | 0.01 | 0.02 |
| **Shijiazhuang** | 2009 | 0.10 | 0.05 | 0.04 |
| **Changsha** | 2009 | 0.09 | 0.04 | 0.04 |
| **Wuhan** | 2009 | 0.11 | 0.04 | 0.05 |
| **Hefei** | 2009 | 0.11 | 0.02 | 0.03 |
| **Taiyuan** | 2009 | 0.11 | 0.08 | 0.02 |
| **Nanchang** | 2009 | 0.08 | 0.05 | 0.04 |
| **Zhengzhou** | 2009 | 0.10 | 0.05 | 0.05 |
| **Shenyang** | 2009 | 0.11 | 0.06 | 0.04 |
| **Changchun** | 2009 | 0.09 | 0.03 | 0.04 |
| **Harbin** | 2009 | 0.10 | 0.05 | 0.05 |
| **Hohhot** | 2009 | 0.07 | 0.05 | 0.04 |
| **Urumqi** | 2009 | 0.14 | 0.09 | 0.07 |
| **Yinchuan** | 2009 | 0.09 | 0.04 | 0.03 |
| **Chengdu** | 2009 | 0.11 | 0.04 | 0.06 |
| **Xi'an** | 2009 | 0.11 | 0.05 | 0.05 |
| **Kunming** | 2009 | 0.07 | 0.04 | 0.05 |
| **Lanzhou** | 2009 | 0.15 | 0.06 | 0.04 |
| **Guiyang** | 2009 | 0.07 | 0.06 | 0.03 |
| **Xining** | 2009 | 0.14 | 0.04 | 0.03 |
| **Nanning** | 2009 | 0.05 | 0.03 | 0.03 |
| **Chongqing** | 2009 | 0.11 | 0.05 | 0.04 |
| **Lhasa** | 2009 | 0.05 | 0.01 | 0.02 |
| **Guangzhou** | 2010 | 0.07 | 0.03 | 0.05 |
| **ShangHai** | 2010 | 0.08 | 0.03 | 0.05 |
| **Beijing** | 2010 | 0.12 | 0.03 | 0.06 |
| **Tianjin** | 2010 | 0.10 | 0.05 | 0.05 |
| **Hangzhou** | 2010 | 0.10 | 0.03 | 0.06 |
| **Nanjing** | 2010 | 0.11 | 0.04 | 0.05 |
| **Jinan** | 2010 | 0.12 | 0.05 | 0.03 |
| **Fuzhou** | 2010 | 0.07 | 0.01 | 0.03 |
| **Haikou** | 2010 | 0.04 | 0.01 | 0.02 |
| **Shijiazhuang** | 2010 | 0.10 | 0.05 | 0.04 |
| **Zhengzhou** | 2009 | 0.10 | 0.05 | 0.05 |
| **Changsha** | 2010 | 0.08 | 0.04 | 0.05 |
| **Wuhan** | 2010 | 0.11 | 0.04 | 0.06 |
| **Hefei** | 2010 | 0.12 | 0.02 | 0.03 |
| **Taiyuan** | 2010 | 0.09 | 0.07 | 0.02 |
| **Nanchang** | 2010 | 0.09 | 0.06 | 0.04 |
| **Zhengzhou** | 2010 | 0.11 | 0.05 | 0.05 |
| **Shenyang** | 2010 | 0.10 | 0.06 | 0.04 |
| **Changchun** | 2010 | 0.09 | 0.03 | 0.04 |
| **Harbin** | 2010 | 0.10 | 0.05 | 0.05 |
| **Hohhot** | 2010 | 0.07 | 0.05 | 0.03 |
| **Urumqi** | 2010 | 0.13 | 0.09 | 0.07 |
| **Yinchuan** | 2010 | 0.09 | 0.04 | 0.03 |
| **Chengdu** | 2010 | 0.10 | 0.03 | 0.05 |
| **Xi'an** | 2010 | 0.13 | 0.04 | 0.05 |
| **Kunming** | 2010 | 0.07 | 0.04 | 0.05 |
| **Lanzhou** | 2010 | 0.16 | 0.06 | 0.05 |
| **Guiyang** | 2010 | 0.08 | 0.06 | 0.03 |
| **Xining** | 2010 | 0.12 | 0.04 | 0.03 |
| **Nanning** | 2010 | 0.07 | 0.03 | 0.03 |
| **Chongqing** | 2010 | 0.10 | 0.05 | 0.04 |
| **Lhasa** | 2010 | 0.05 | 0.01 | 0.02 |
| **Guangzhou** | 2011 | 0.07 | 0.03 | 0.05 |
| **ShangHai** | 2011 | 0.08 | 0.03 | 0.05 |
| **Beijing** | 2011 | 0.11 | 0.03 | 0.06 |
| **Tianjin** | 2011 | 0.09 | 0.04 | 0.04 |
| **Hangzhou** | 2011 | 0.09 | 0.04 | 0.06 |
| **Nanjing** | 2011 | 0.10 | 0.03 | 0.05 |
| **Jinan** | 2011 | 0.10 | 0.05 | 0.04 |
| **Fuzhou** | 2011 | 0.07 | 0.01 | 0.03 |
| **Haikou** | 2011 | 0.04 | 0.01 | 0.02 |
| **Shijiazhuang** | 2011 | 0.10 | 0.05 | 0.04 |
| **Changsha** | 2011 | 0.08 | 0.04 | 0.05 |
| **Wuhan** | 2011 | 0.10 | 0.04 | 0.06 |
| **Hefei** | 2011 | 0.11 | 0.02 | 0.03 |
| **Taiyuan** | 2011 | 0.08 | 0.06 | 0.02 |
| **Nanchang** | 2011 | 0.09 | 0.06 | 0.04 |
| **Zhengzhou** | 2011 | 0.10 | 0.05 | 0.05 |
| **Shenyang** | 2011 | 0.10 | 0.06 | 0.03 |
| **Changchun** | 2011 | 0.09 | 0.03 | 0.04 |
| **Harbin** | 2011 | 0.10 | 0.04 | 0.05 |
| **Hohhot** | 2011 | 0.08 | 0.05 | 0.04 |
| **Urumqi** | 2011 | 0.13 | 0.08 | 0.07 |
| **Yinchuan** | 2011 | 0.10 | 0.04 | 0.03 |
| **Chengdu** | 2011 | 0.10 | 0.03 | 0.05 |
| **Xi'an** | 2011 | 0.12 | 0.04 | 0.04 |
| **Kunming** | 2011 | 0.06 | 0.04 | 0.04 |
| **Lanzhou** | 2011 | 0.14 | 0.05 | 0.04 |
| **Guiyang** | 2011 | 0.08 | 0.05 | 0.03 |
| **Xining** | 2011 | 0.10 | 0.04 | 0.03 |
| **Nanning** | 2011 | 0.07 | 0.03 | 0.03 |
| **Chongqing** | 2011 | 0.09 | 0.04 | 0.03 |
| **Lhasa** | 2011 | 0.04 | 0.01 | 0.02 |
| **Guangzhou** | 2012 | 0.07 | 0.02 | 0.05 |
| **ShangHai** | 2012 | 0.07 | 0.02 | 0.05 |
| **Beijing** | 2012 | 0.11 | 0.03 | 0.05 |
| **Tianjin** | 2012 | 0.11 | 0.05 | 0.04 |
| **Hangzhou** | 2012 | 0.09 | 0.04 | 0.05 |
| **Nanjing** | 2012 | 0.10 | 0.03 | 0.05 |
| **Jinan** | 2012 | 0.10 | 0.06 | 0.04 |
| **Fuzhou** | 2012 | 0.06 | 0.01 | 0.04 |
| **Haikou** | 2012 | 0.03 | 0.01 | 0.02 |
| **Shijiazhuang** | 2012 | 0.10 | 0.06 | 0.04 |
| **Changsha** | 2012 | 0.09 | 0.03 | 0.04 |
| **Wuhan** | 2012 | 0.10 | 0.03 | 0.05 |
| **Hefei** | 2012 | 0.10 | 0.02 | 0.03 |
| **Taiyuan** | 2012 | 0.08 | 0.06 | 0.03 |
| **Nanchang** | 2012 | 0.09 | 0.05 | 0.04 |
| **Zhengzhou** | 2012 | 0.11 | 0.05 | 0.05 |
| **Shenyang** | 2012 | 0.09 | 0.06 | 0.04 |
| **Changchun** | 2012 | 0.09 | 0.03 | 0.04 |
| **Harbin** | 2012 | 0.09 | 0.04 | 0.05 |
| **Hohhot** | 2012 | 0.09 | 0.05 | 0.04 |
| **Urumqi** | 2012 | 0.15 | 0.06 | 0.07 |
| **Yinchuan** | 2012 | 0.10 | 0.04 | 0.04 |
| **Chengdu** | 2012 | 0.12 | 0.03 | 0.05 |
| **Xi'an** | 2012 | 0.12 | 0.04 | 0.04 |
| **Kunming** | 2012 | 0.07 | 0.03 | 0.04 |
| **Lanzhou** | 2012 | 0.14 | 0.04 | 0.04 |
| **Guiyang** | 2012 | 0.07 | 0.03 | 0.03 |
| **Xining** | 2012 | 0.11 | 0.04 | 0.03 |
| **Nanning** | 2012 | 0.07 | 0.02 | 0.03 |
| **Chongqing** | 2012 | 0.09 | 0.04 | 0.04 |
| **Lhasa** | 2012 | 0.05 | 0.01 | 0.02 |

The data in 2003-2006 of Lhasa was not included in calculation, because GDP per capita for Lhasa could not be calculated without corresponding demographic statistics for these years. All the data was retrieved from the National Bureau of Statistics of China.
